# Hepatoprotective effect of taxifolin on cyclophosphamide-induced oxidative stress, inflammation, and apoptosis in mice: Involvement of Nrf2/HO-1 signaling

**DOI:** 10.17305/bb.2022.8743

**Published:** 2023-08-01

**Authors:** Osama Y Althunibat, Mohammad H Abukhalil, Muthana M Jghef, Manal A Alfwuaires, Abdulmohsen I Algefare, Bader Alsuwayt, Reem Alazragi, Mohammed A S Abourehab, Afaf F Almuqati, Shaik Karimulla, Saleem H Aladaileh

**Affiliations:** 1 Department of Medical Analysis, Princess Aisha Bint Al-Hussein College of Nursing and Health Sciences, Al-Hussein Bin Talal University, Ma’an, Jordan; 2 Department of Biology, College of Science, Al-Hussein Bin Talal University, Ma’an, Jordan; 3 Department of Radiology, College of Medical Technology, Al-Kitab University, Kirkuk, Iraq; 4 Department of Biological Sciences, Faculty of Science, King Faisal University, Al-Ahsa, Saudi Arabia; 5 Department of Pharmacy Practice, College of Pharmacy, University of Hafr Al-Batin, Hafr Al-Batin, Saudi Arabia; 6 Department of Biochemistry, College of Science, University of Jeddah, Jeddah, Saudi Arabia; 7 Department of Pharmaceutics, College of Pharmacy, Umm Al-Qura University, Makkah, Saudi Arabia; 8 Department of Pharmaceutical Chemistry, College of Pharmacy, University of Hafr Al-Batin, Hafr Al-Batin, Saudi Arabia

**Keywords:** Flavonoids, cyclophosphamide (CP), inflammation, taxifolin (TA), hepatotoxicity, oxidative damage, nuclear factor erythroid 2-related factor 2 (Nrf2)

## Abstract

Taxifolin (TA) is a natural flavonoid found in many foods and medicinal plants with well-documented antioxidant and anti-inflammatory properties. Cyclophosphamide (CP) is an effective antineoplastic and immunosuppressive agent; however, it is associated with numerous adverse events, including hepatotoxicity. Herein, we aimed to investigate the potential protective effects of TA using a mouse model of CP-induced hepatotoxicity. Mice were co-treated with TA (25 and 50 mg/kg, orally) and CP (30 mg/kg, i.p.) for 10 consecutive days and sacrificed 24 hours later. CP induced increased transaminases (ALT and AST), alkaline phosphatase (ALP), and lactate dehydrogenase (LDH) paralleled with pronounced histopathological alterations in the liver. Moreover, hepatic tissues of CP-injected mice showed increased malondialdehyde (MDA), protein carbonyl, and nitric oxide (NO) levels, accompanied by decreased antioxidant defenses (glutathione [GSH], superoxide dismutase [SOD], and catalase [CAT]). Livers of CP-injected mice also showed increased inflammatory response (nuclear transcription factor kappa-B [NF-κB] p65 activation, increased levels of proinflammatory cytokines tumor necrosis factor alpha [TNF-α], interleukin 1 beta [IL-1β], and IL-6) and apoptosis (decreased Bcl-2 and increased Bax and caspase-3 expression levels). Remarkably, TA ameliorated markers of liver injury and histological damage in CP-injected mice. TA treatment also attenuated numerous markers of oxidative stress, inflammation, and apoptosis in the liver of CP-injected mice. This was accompanied by increased nuclear factor erythroid 2-related factor 2 (Nrf2)/heme oxygenase 1 (HO-1) expression in the liver tissues of CP-injected mice. Taken together, this study indicates that TA may represent a promising new avenue to prevent/treat CP-induced hepatotoxicity and perhaps other liver diseases associated with oxidative stress and inflammation.

## Introduction

The alkylating agent cyclophosphamide (CP) is one of the most widely utilized chemotherapeutic and immunosuppressive agents [1, 2]. However, it is associated with multiple toxic side effects, including hepatotoxicity, cardiotoxicity, nephrotoxicity, and testicular toxicity, restricting its clinical use [3–8]. Although the exact cellular mechanisms by which CP induces hepatotoxicity are poorly understood, several studies have demonstrated that oxidative stress, inflammatory response, and cell death are key components in triggering and driving the pathological events associated with CP hepatotoxicity [5, 6, 9, 10]. Indeed, metabolic conversion of CP by hepatic microsomal cytochrome P-450 mixed function oxidase system results in the formation of phosphoramide mustard and acrolein [11, 12]. The highly reactive metabolite acrolein is thought to be a main culprit of CP-induced hepatotoxicity [13, 14]. It can contribute to increased generation of reactive oxygen species (ROS) and results in dysfunction of important antioxidant defense mechanisms, leading to lipid peroxidation, oxidative protein modifications, DNA damage and, consequently, oxidative tissue injury and liver dysfunction [15–17]. The persistently-enhanced ROS production may prompt the activation of different proinflammatory pathways, such as nuclear transcription factor kappa-B (NF-κB), eventually culminating in hepatic inflammation and cell death [5, 6, 18–20]. Therefore, optimal therapeutic approaches for protection against CP hepatotoxicity and other complications are needed. Several preclinical studies showed that naturally occurring compounds have demonstrated effective therapeutic benefits against CP-induced organs damage through attenuation of tissue inflammation and oxidative damage and activation of nuclear factor erythroid 2-related factor 2 (Nrf2) [5, 6, 9, 21–23].

**Figure 1.**
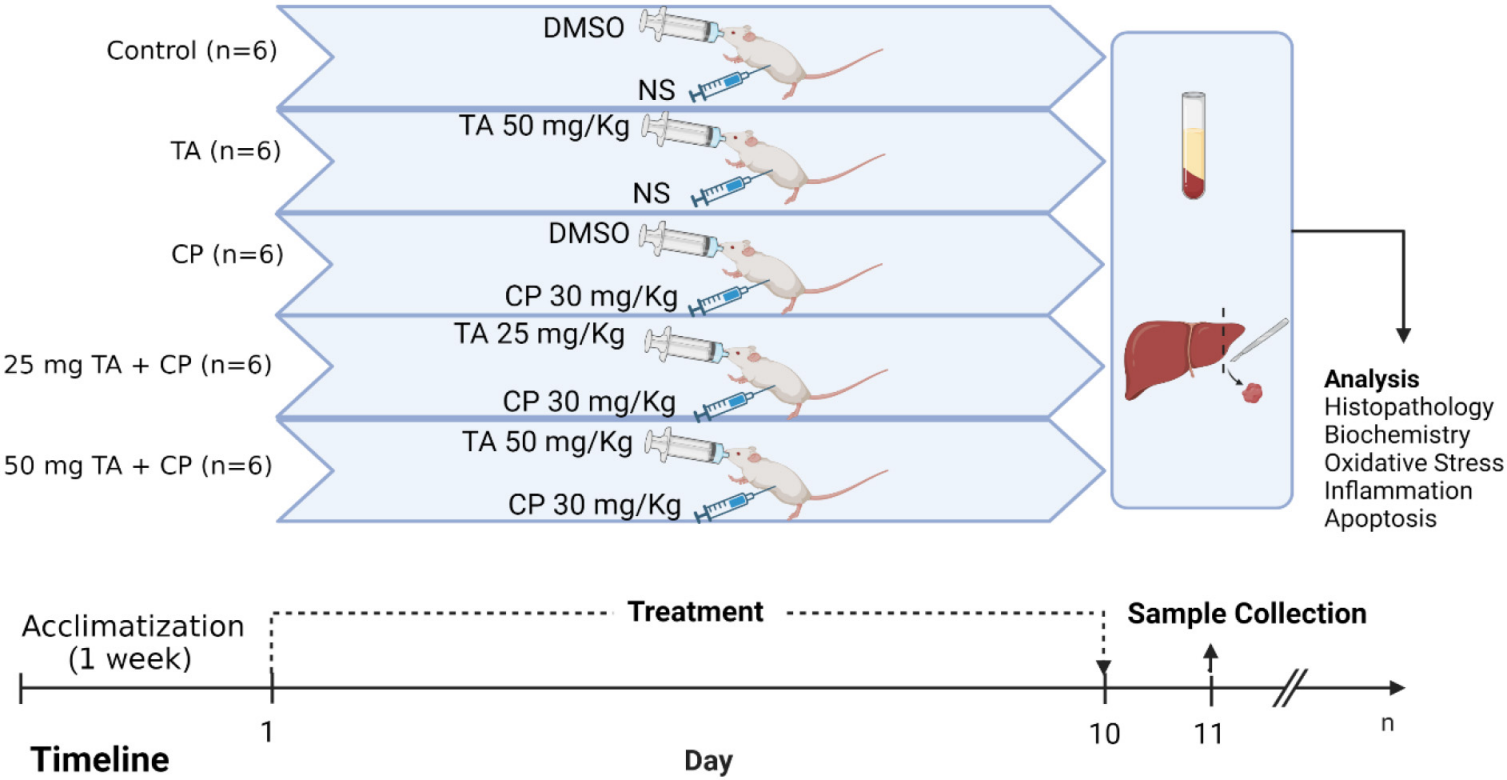
**Overview of experimental design.** TA: Taxifolin; CP: Cyclophosphamide; DMSO: Dimethyl sulfoxide; NS: Normal saline.

Plants and their bioactive compounds have attracted considerable attention in the medical industry due to their safety and therapeutic effects [24]. Among the medically relevant plant-based active compounds, polyphenols, such as flavonoids, are secondary metabolites of plants with various potential health-promoting benefits, including antioxidant, anti-inflammatory, antibacterial, and anticancer properties [25–29]. Taxifolin (TA; dihydroquercetin) is a bioactive flavonoid commonly found in grapes, olive oil, citrus fruits, onions, and conifers with well-recognized pharmacological actions, including antioxidant, anti-inflammatory effects [30–32], in addition to its anticancer [33], hepatoprotective [34], and antimicrobial [35] properties. It has been demonstrated that TA showed a hepatoprotective effect in a mouse model of acute alcohol-induced hepatic injury by attenuation of oxidative stress, NF-κB-mediated inflammation, and apoptosis and modulation of PI3K/Akt signaling pathway [36]. Moreover, TA effectively inhibited concanavalin A-mediated immunological hepatic injury in mice by attenuating proinflammatory cytokines expression and CD4+ and CD8+ T cells infiltration in the hepatic tissues and prevented tumor necrosis factor alpha (TNF-α)/actinomycin D (Act D)-induced apoptosis in HepG2 cells by inhibiting the activation of caspases [37]. TA has also been shown to prevent cadmium-induced renal damage [31] and to attenuate cardiotoxicity caused by isoproterenol [32] through modulating oxidative damage, inflammatory response, and cell apoptosis. Tissue protective potential of TA has also been attributed to its upregulating effect on Nrf2/heme oxygenase 1 (HO-1) signaling pathway. Nrf2 plays a crucial role in maintaining redox homeostasis by robustly activating the transcription of multiple antioxidant and cytoprotective enzymes [38]. Accumulating evidence demonstrates that Nrf2 activation may have tremendous protective actions in tissue injury that is largely associated with oxidative stress and inflammation [6, 31, 32, 39].

Despite its tremendous treatment potential toward multiple diseases, the protective effect of TA on CP-induced hepatotoxicity has not been studied. Herein, we tested the hypothesis that TA treatment would protect mice from CP-induced hepatotoxicity by decreasing oxidative damage, inflammation, and apoptosis, improving antioxidant defenses, and boosting Nrf2/HO-1 signaling in the liver. This study describes a potent role of TA in reducing the extent of CP-induced hepatocellular damage, oxidative stress, inflammation, and cell death in the liver.

## Materials and methods

### Animals and study design

The study included 30 Swiss albino mice (22–26 g), which were housed under constant temperature (23 ± 2 ^∘^C) and humidity, with 12-h alternating light and dark cycles while they were given free access to food and water.

As illustrated in Figure 1, mice were divided into 5 groups (*n* ═ 6) and accommodated in separate cages. They were left for a week to acclimatize to the experiment conditions before starting the experiment. The treatment procedure involves co-treatment of 2 animal groups with CP (30 mg/kg, i.p) and TA (25 or 50 mg/kg, P.O) for 10 consecutive days, while the other 3 groups were administered with physiological saline (i.p) and 5% dimethyl sulfoxide (DMSO) (P.O) (control), TA (50 mg/kg, P.O), or CP (30 mg/kg, i.p) throughout the experiment period. CP injection (Endoxan^®^ , Baxter Oncology, Halle, Germany) and TA (Biosynth Carbosynth, UK) were freshly dissolved in physiological saline and 5% DMSO, respectively. CP dose and treatment regimen were selected according to the previous literature [10], while TA doses were chosen based on previous works that showed the ability of TA to upregulate Nrf2/HO-1 signaling pathway and to attenuate the oxidative tissue injury and key regulators of inflammatory response and apoptosis without showing any toxicity [31, 32, 40].

On day 11, the animals were sacrificed after induction of anesthesia by intraperitoneal injection of xylazine (10 mg/kg) and ketamine (100 mg/kg) combination, and blood sample collection via cardiac puncture. Then, the mice were dissected, and livers were immediately obtained and washed with cold phosphate buffer saline (PBS, 50 mM, pH 7.0) and cut into portions. Parts of the liver tissues were fixed in 10% neutral buffered formalin (NBF) for histopathological and immunohistochemistry (IHC) analysis, while other parts were homogenized in cold PBS (1:10 w/v) using Omni general laboratory homogenizer (GLH-850, Omni International, Kennesaw, GA, USA). The homogenates were then centrifuged, and the supernatant was collected and deep frozen in aliquots for further biochemical and molecular investigations. Blood samples were centrifuged, and serum was aliquoted in the deep freezer for liver enzymes assessments.

### Histological evaluation of tissue damage

The histopathological examination of liver tissues was performed after the preparation hematoxylin and eosin (H&E) stained histological slides. For this purpose, the NBF fixed tissues were dehydrated followed by embedding the dehydrated tissues in paraffin and preparation of 5-micron thickness slices. The serial sections were subjected to H&E staining [41]. The prepared slides were then evaluated by a histopathologist under a light microscope.

### Serum levels of liver enzymes

Activities of transaminases (AST and ALT), alkaline phosphatase (ALP), and lactate dehydrogenase (LDH) in serum samples were investigated by spectrophotometry using commercial kits purchased from Spectrum Diagnostics, Egypt, according to the manufacturer’s instructions.

### Hepatic oxidative stress markers and antioxidants

The level of hepatic malondialdehyde (MDA), a marker of lipid peroxidation, was determined following its reaction with thiobarbituric acid (TBA) in acidic medium resulting in the formation of TBA reactive products [42]. The level of protein carbonyl in the liver homogenates was measured based on its reaction with 2,4-dinitrophenylhydrazine (DNPH) to produce dinitrophenyl (DNP) hydrazine [43]. The hepatic contents of nitric oxide (NO) were evaluated by measurement of nitrite produced diazotize sulfanilamide upon undergoing in Gries reaction [44].

Furthermore, contents of reduced glutathione (GSH) and glutathione disulfide (GSSG) were assessed based on the capability of GSH to reduce 5, 5 dithiobis(2-nitrobenzoic acid) (DNTB) into 5-thio-2-nitrobenzoic acid (TNB) according to the method described by Griffith [45]. Hence, GSH/GSSG ratio was calculated. Moreover, the superoxide dismutase (SOD) activity in liver homogenates was assessed based on its potential to hinder nitro blue tetrazolium (NBT) dye reduction to blue formazan by superoxide anion [46]. Catalase (CAT) activity in liver homogenate samples was investigated based on its capacity to decompose hydrogen peroxide (H_2_O_2_) into water and oxygen [47]. Finally, an ELISA kit (FineTest, Wuhan, Hubei, China) was used to determine HO-1 levels in the liver according to the manufacturer’s instructions.

### Hepatic proinflammatory cytokines

The quantitative determination of TNF-α, interleukin 1 beta (IL-1β), and IL-6 levels were determined using ELISA kits provided by FineTest (Wuhan, Hubei, China), according to the manufacturer’s instructions.

### Liver immunohistochemistry

The IHC staining was employed to investigate the expression levels of inflammatory mediator (NF-κB p65), pro-apoptotic (BAX and caspase-3), anti-apoptotic (Bcl-2), and antioxidant regulator (Nrf2) proteins in liver tissues. Summarily, the remaining deparaffinized liver sections were subjected to antigen retrieval process through consecutive treatment with 50 mM citrate buffer (pH 6.8) and 0.3% H_2_O_2_. Nonspecific antigen–antibody binding was blocked by applying the normal serum for 20 min. Then, the slices were washed with PBS followed by incubation with primary antibodies against the target proteins overnight at 4 ^∘^C, with the following specifications: anti-NF-κB p65 antibodies (Santa Cruz Biotechnology, Dallas, TX, USA; 1:100 dilution), anti-Bax antibodies (Invitrogen, Waltham, MA, USA; 1:100 dilution), anti-Bcl-2 antibodies (Invitrogen, Waltham, MA, USA; 1:50 dilution), anti-caspase-3 antibodies (Invitrogen, Waltham, MA, USA; 1:100 dilution), and anti-Nrf2 antibodies (Invitrogen, Waltham, MA, USA; 1:100 dilution). After washing the unbound primary antibodies, sections were incubated with the conjugated secondary antibodies. A goat anti-rabbit secondary antibody (EnVision+™ System Horseradish Peroxidase Labelled Polymer) for polyclonal antibodies (anti-caspase 3, anti-NF-κB p65, and anti-Nrf2), and mouse monoclonal secondary antibody (EnVision+™ System Horseradish Peroxidase Labelled Polymer) for monoclonal antibodies (anti-Bcl-2 and anti-Bax), provided by Dako (Santa Clara, CA, USA) were used. Further, the substrate DAB was then applied for color development followed by adding the counterstain (Mayer’s hematoxylin). The slides were then evaluated under a light microscope provided with capturing camera. Assessment of the staining intensity of immunostaining for NF-κB p65, Bax, and caspase-3 was presented as a percentage of positive expression in total 1000 cells per 8 high-power field while immunostaining for Bcl-2 and Nrf2 was determined through the area of positive expression using ImageJ analysis software (NIH, Bethesda, MD, USA).

### Ethical statement

All animal experiments reported in this research complied with the National Institutes of Health standards (NIH publication No. 85-23, revised 2011) and were approved by the Research Ethics Committee of the King Faisal University (KFU-REC-2022-ETHICS333).

**Figure 2.**
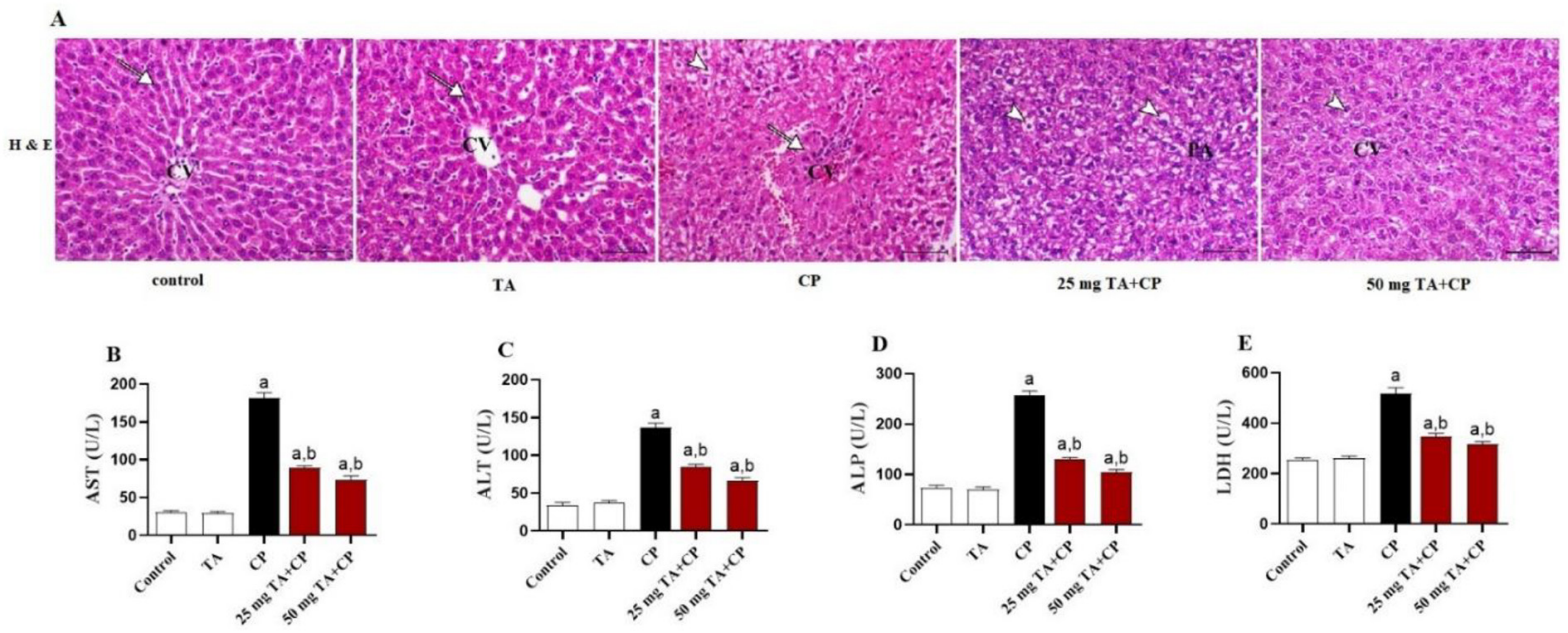
**TA mitigates the CP-induced liver injury in mice.** (A) Representative H&E staining of liver tissue sections from control and TA-treated mice demonstrating normal hepatocytes arranged in cords, around a central vein (CV indicates central vein and arrow indicates hepatocytes); CP-injected mice showing centrilobular hepatic necrosis (arrowhead) associated with hepatic vacuolation (arrowhead) (CV indicates central vein); CP-injected mice co-treated with 25 mg TA showing moderate degree of hepatic vacuolation extending from the periportal area (PA) (arrowheads); and CP-injected mice co-treated with 50 mg TA showing mild degree of hepatic vacuolation (arrowhead) (CV indicates central vein), (H&E, X200; Scale bar, 50 µm). (B–E) Serum levels of liver injury biomarkers are displayed, including AST (B), ALT (C), ALP (D), and LDH (E). Values are mean ± S.E.M. (*n* ═ 6 per group). a: *P* < 0.05 vs control; b: *P* < 0.05 vs CP. TA: Taxifolin; H&E: Hematoxylin and eosin; CP: Cyclophosphamide; ALP: Alkaline phosphatase; LDH: Lactate dehydrogenase; AST: Aspartate transaminase; ALT: Alanine transaminase.

### Statistical analysis

Values are expressed as the mean ± standard error of the mean (S.E.M.). For the statistical comparison between groups, the Graph Pad Prism software GraphPad Prism 7 software (San Diego, CA, USA) was employed. Data were compared by one-way analysis of variance (ANOVA) followed by Tukey’s post-hoc test. The significance level was set at *P* < 0.05.

## Results

### The CP-induced liver injury and histopathological alterations are prevented by TA

The hepatoprotective effect of TA on CP-induced liver injury was assessed by evaluation of histological changes in liver tissues prepared with H&E staining and determination of serum indicators of liver injury, including transaminases, ALP, and LDH. Histopathological analysis of H&E-stained hepatic sections from control and TA-treated groups showed normal hepatocytes arranged in cords, around a central vein (Figure 2A). Analysis of H&E-stained liver sections of CP-treated mice demonstrated marked centrilobular hepatic necrosis associated with hepatic vacuolation (Figure 2A). In addition to that, CP-treated mice showed a significant (*P* < 0.05) increase in serum AST, ALT, ALP, and LDH levels as compared to control mice (Figure 2B–2D). TA treatment significantly prevented liver injury as evidenced by the attenuation of histopathological changes (Figure 2A) and the amelioration of the increased AST, ALT, ALP, and LDH values in serum (Figure 2B–2E). The TA treatment alone had no effect in healthy animals on all variables studied.

### The CP-induced hepatic oxidative stress is attenuated by TA

**Figure 3.**
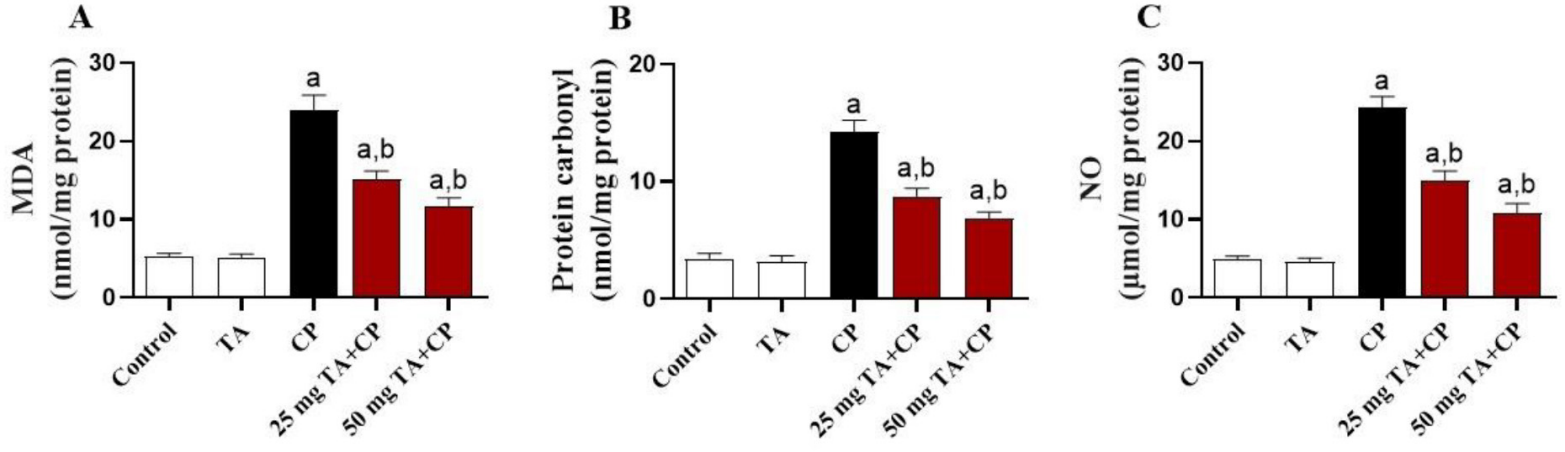
**CP-induced hepatic oxidative damage is ameliorated by TA in mice.** Hepatic levels of oxidative stress markers are displayed, including (A) MDA, (B) protein carbonyl, and (C) NO in indicated groups. Values are mean ± standard error of the mean (S.E.M.) (*n* ═ 6 per group). a: *P* < 0.05 vs control; b: *P* < 0.05 vs CP. MDA: Malondialdehyde; NO: Nitric oxide; CP: Cyclophosphamide; TA: Taxifolin.

**Figure 4.**
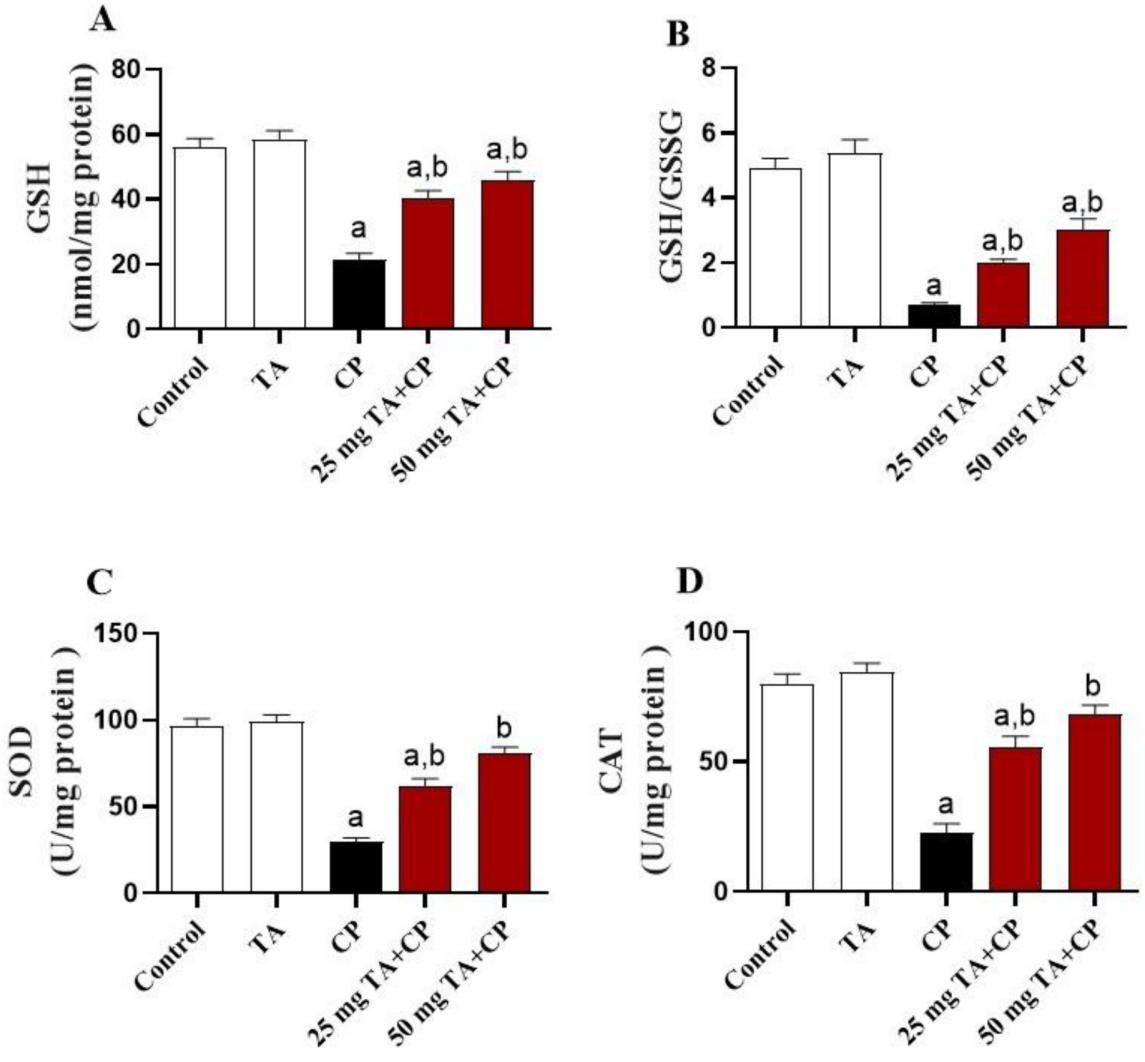
**TA enhances antioxidants in the liver of CP-injected mice.** Hepatic antioxidants are presented, including (A) GSH level, (B) GSH/GSSG ratio, and (C) SOD and (D) CAT activities in each study group. Values are mean ± standard error of the mean (S.E.M.) (*n* ═ 6 per group). a: *P* < 0.05 vs control; b: *P* < 0.05 vs CP. TA: Taxifolin; CP: Cyclophosphamide; GSH: Glutathione; GSSG: Glutathione disulfide; SOD: Superoxide dismutase; CAT: Catalase.

Oxidative stress plays a key role in the pathomechanism of CP-induced liver injury. Compared to control animals, CP significantly (*P* < 0.05) increased the hepatic levels of MDA, protein carbonyl, and NO (Figure 3A–3C), in parallel with a significant (*P* < 0.05) reduction in GSH contents, GSH/GSSG ratio, and SOD and CAT activities in the liver (Figure 4A–4D). The CP-induced increased oxidative stress was significantly (*P* < 0.05) attenuated by TA (25 and 50 mg/kg) treatment. The TA by itself exerted no effect in the liver of healthy animals on any of the abovementioned variables.

### The CP-induced hepatic inflammation is mitigated by TA

**Figure 5.**
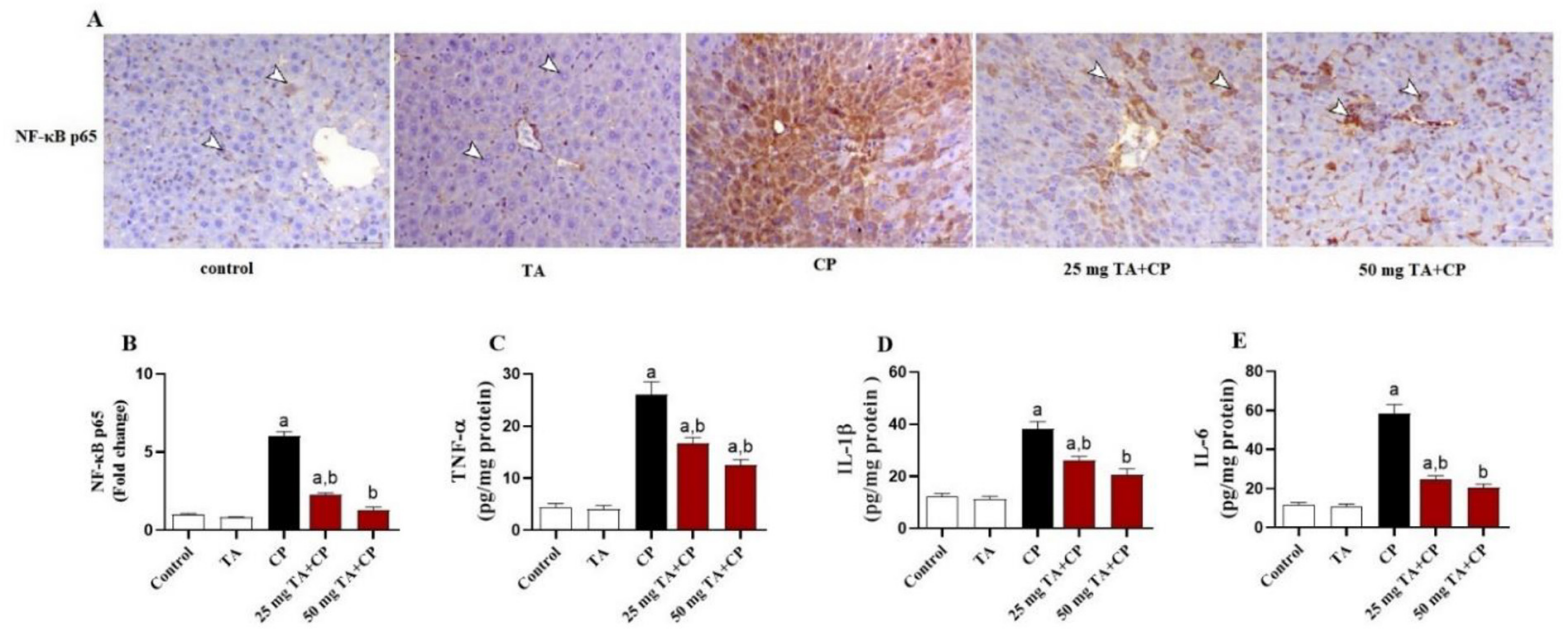
**Hepatic inflammation in CP-injected mice is attenuated by the treatment with TA.** (A) Representative images of the hepatic NF-κB p65 immunostaining from indicated groups are displayed (Brown represents increases of NF-κB p65 staining intensity in CP groups; IHC, X200; Scale bar, 50 µm); (B) Quantification of NF-κB p65 immunostaining in indicated groups expressed as fold change of the control group; (C–E) Hepatic levels of TNF-α (C), IL-1β (D), and IL-6 (E) in indicated groups. Values are mean ± standard error of the mean (S.E.M.) (*n* ═ 6 per group). a: *P* < 0.05 vs control; b: *P* < 0.05 vs CP. CP: Cyclophosphamide; TA: Taxifolin; NF-κB: Nuclear transcription factor kappa-B; IHC: Immunohistochemistry; TNF-α: Tumor necrosis factor alpha; IL-1β: Interleukin 1 beta.

Because NF-κB p65 activation and proinflammatory cytokines production are critical mediators of liver injury after CP exposure, we studied the effects of TA on inflammatory response as well. Herein, CP induced a notable (*P* < 0.05) increase in hepatic NF-κB p65 expression and in the levels of the proinflammatory cytokines, TNF-α, IL-1β, and IL-6 in the liver (Figure 5A–5E). Remarkably, TA (25 and 50 mg/kg) treatment exerted an anti-inflammatory effect in the liver of CP-treated mice, as evidenced by both mitigation of the NF-κB p65 protein expression (Figure 5A and 5B) and significant reduction of the levels of TNF-α, IL-1β, and IL-6 (Figure 5C–5E). The TA by itself had no effect on the abovementioned variables in the liver of healthy animals.

### The CP-induced apoptosis in mouse liver is suppressed by TA

**Figure 6.**
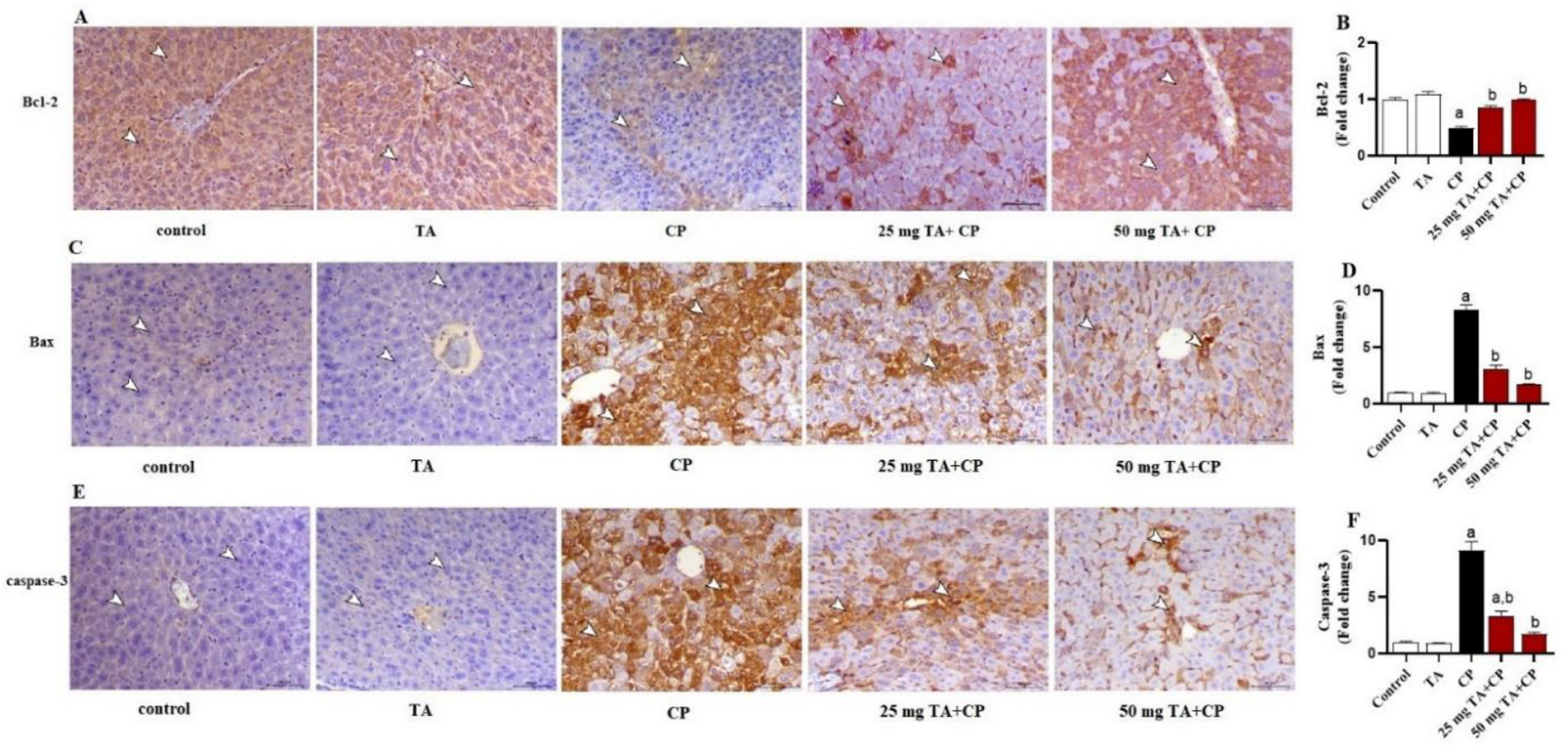
**CP-induced apoptosis in mouse liver is attenuated by the treatment of TA.** (A) Representative images of the hepatic Bcl-2 immunostaining from indicated groups are displayed (brown represents increases of Bcl-2 staining intensity in CP groups; IHC, X200; Scale bar, 50 µm); (B) Quantification of Bcl-2 immunostaining expressed as fold change of the control group; (C) Representative images of the hepatic Bax immunostaining from indicated groups are displayed (brown represents increases of Bax staining intensity in CP groups; IHC, X200; Scale bar, 50 µm); (D) Quantification of Bax immunostaining expressed as fold change of the control group; (E) Representative images of the hepatic caspase-3 immunostaining from indicated groups are displayed (brown represents increases of caspase-3 staining intensity in CP groups; IHC, X200; Scale bar, 50 µm); (F) Quantification of caspase-3 immunostaining expressed as fold change of the control group. Values are mean ± standard error of the mean (S.E.M.) (*n* ═ 6 per group). a: *P* < 0.05 vs control; b: *P* < 0.05 vs CP. CP: Cyclophosphamide; TA: Taxifolin; IHC: Immunohistochemistry.

To further investigate the efficacy of TA on CP-induced liver injury in mice, we assessed hepatic expression levels of apoptosis regulatory proteins, such as Bax, Bcl-2, and caspase-3. CP greatly (*P* < 0.05) decreased Bcl-2 protein expression level, paralleled with a significant (*P* < 0.05) increase in hepatic Bax and caspase-3 expression (Figure 6A–6F). Intriguingly, the CP-induced apoptosis in the liver was attenuated by TA (25 and 50 mg/kg) treatment (Figure 6A–6F). However, the hepatic apoptosis markers were unaffected by the administration of TA alone in healthy mice.

### TA upregulates Nrf2/HO-1 in mouse liver

**Figure 7.**
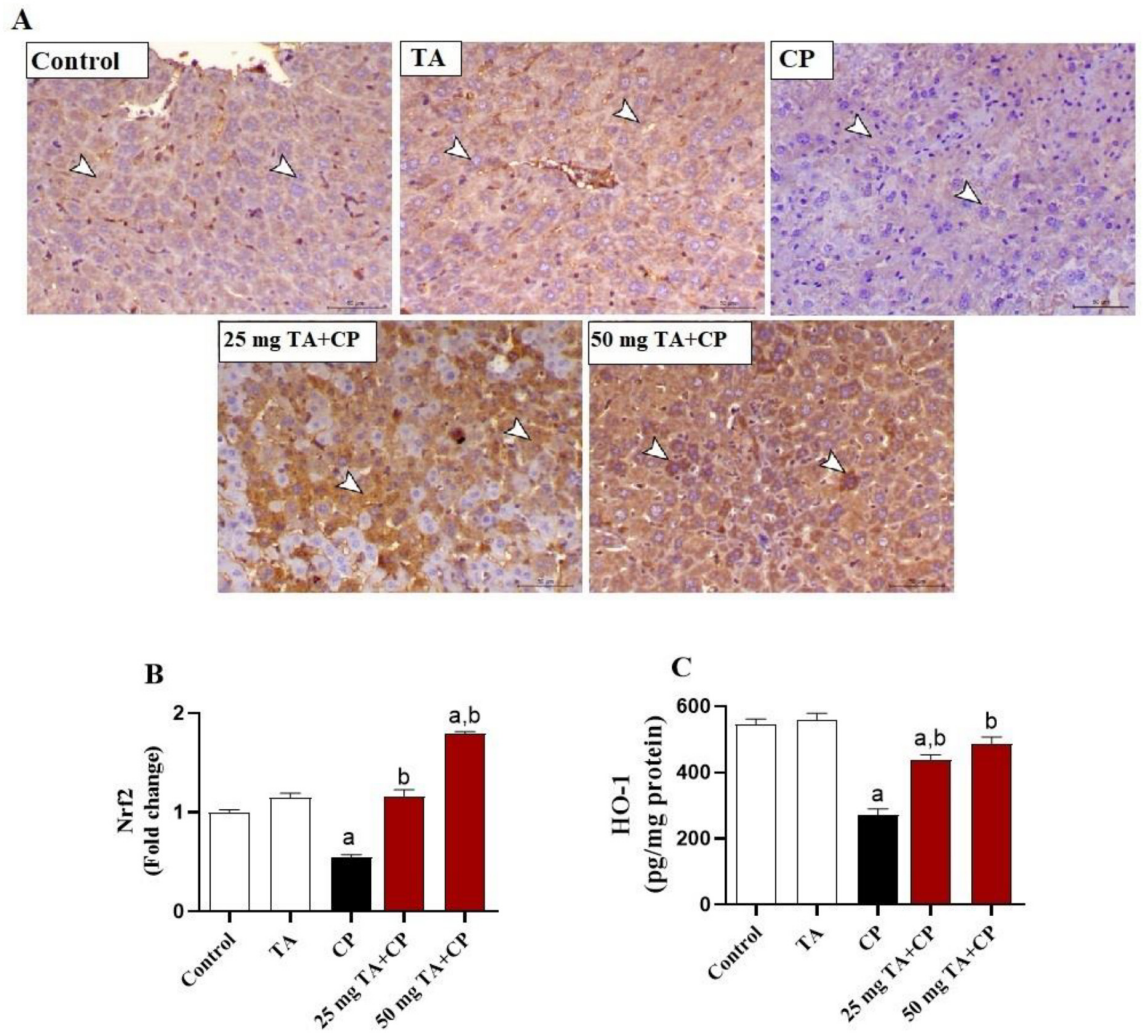
**Hepatic Nrf2/HO-1 pathway is upregulated by TA in CP-injected mice.** (A) Representative images of the hepatic Nrf2 immunostaining from indicated groups are displayed (brown represents increases of Nrf2 staining intensity in CP groups; IHC, X200; Scale bar, 50 µm); (B) Quantification of Nrf2 immunostaining in indicated groups expressed as fold change of the control group; (C) Hepatic levels of HO-1 in indicated groups are measured by ELISA. Values are mean ± standard error of the mean (S.E.M.) (*n* ═ 6 per group). a: *P* < 0.05 vs control; b: *P* < 0.05 vs CP. Nrf2: Nuclear factor erythroid 2-related factor 2; HO-1: Heme oxygenase 1; TA: Taxifolin; CP: Cyclophosphamide; IHC: Immunohistochemistry.

To explore the molecular background of the protective effects of TA on CP-induced hepatotoxicity, we evaluated Nrf2 protein expression (Figure 7A and 7B) and HO-1 levels (Figure 7C) in the liver. In livers of CP-treated mice, significantly (*P* < 0.05) decreased Nrf2 protein expression and HO-1 levels were observed as compared to the control animals. Such downregulation of Nrf2 and HO-1 in the liver of CP-treated mice was significantly (*P* < 0.05) attenuated by TA (25 and 50 mg/kg) treatment (Figure 7A–7C). TA treatment exerted no effect on hepatic Nrf2 and HO-1 levels in healthy animals.

## Discussion

Multiple lines of evidence suggest that plant-based phytochemicals can be used to treat a variety of diseases due to their various biological activities, including antioxidant, anti-inflammatory, and anticancer properties [24, 36, 48–52]. The present study showed that CP leads to increased hepatic oxidative damage, inflammation, and apoptosis, which are major driving forces of CP hepatotoxicity [5, 6, 22, 23]. Intriguingly, we demonstrate that TA by attenuating tissue oxidative stress, inflammation, and fibrosis and by activating Nrf2/HO-1 signaling significantly prevents liver injury in a mouse model of CP hepatotoxicity.

It has been reported that CP-induced hepatotoxicity is commonly demonstrated by several histopathological alterations, including hepatocytes cytoplasmic vacuolation, liver steatosis, infiltration of inflammatory cells in hepatic parenchyma, blood congestion in portal vein, portal fibrosis, and appearance of focal necrotic areas [5, 6, 53, 54]. Furthermore, hepatocyte damage after CP administration is associated with an increase in serum levels of liver function enzymes, such as ALT, AST, ALP, and LDH [5, 6, 55]. Likewise, the development of liver injury following CP administration was demonstrated in the current study by several histopathological alterations and also by increased circulating liver enzymes. Remarkably, TA treatment attenuated CP-induced liver injury, as shown by attenuated histopathological alterations paralleled with decreased circulating levels of serum liver enzymes. Accordingly, the hepatoprotective action of TA against acetaminophen- [56] and carbon tetrachloride (CCl_4_)- [50] induced liver injury was reported.

Although the exact molecular mechanism underlying CP-induced liver toxicity is not fully understood, several studies have been reported that oxidative stress plays a critical role in the pathomechanism of CP-induced liver injury [5, 6, 9, 23]. CP undergoes several oxidations by hepatocytes cytochrome P450 mixed function oxidase enzymes to produce oxidative agents like acrolein which mediates overproduction of free radicals, including ROS and NO. CP-induced free radicals formation induces oxidative alteration of multiple cellular components, culminating in membrane lipid peroxidation, protein nitration, and structural defect of enzymes, including antioxidant enzymes. The cellular status of overwhelmed oxidative agents accompanied by decreased antioxidant contents is described as oxidative stress. As documented by many recent reports [5–7, 10, 18], oxidative stress in liver of CP-injected mice was evidenced in the present study by the increased level of MDA, protein carbonyl, and NO and by decreased GSH levels and antioxidant enzymes (SOD and CAT) activities. Increased lipid peroxidation causes alterations in membrane fluidity and membrane integrity, thus disrupting membrane permeability [57]. Besides, aldehyde derivatives of lipid peroxidation further stimulate cell damage by prompting protein oxidation and nitration leading to structural defect of membrane-bound enzymes as well as aggregation and breakdown of cellular proteins [58]. In addition, oxidative stress may provoke cell death by elevating the cytotoxic intermediate peroxynitrite from the interaction of superoxide anion with NO, further attacks various biomolecules, leading to the dysfunction of critical cellular processes and consequent organ damage [59].

Modulation of oxidative damage and improvement of antioxidant defenses are, therefore, considered one of the important therapeutic strategies for hepatoprotection against the devastating effect of CP. Herein, TA treatment largely attenuated the CP-induced increased hepatic oxidative stress, as evidenced by the notably decreased MDA, protein carbonyl, and NO contents, paralleled with increased SOD, CAT, and GSH in the liver of mice. These findings are in agreement with recent findings in a mouse model of isoproterenol-induced cardiac injury, in which TA decreased MDA and NO contents and increased antioxidants (GSH, SOD, CAT) in the heart [32]. Besides, TA was shown to ameliorate oxidative tissue injury in alcohol- [36] and CCl_4_- [50] induced liver injury and cisplatin-induced nephrotoxicity [48] in mice through suppression of lipid peroxidation and restoration of antioxidant defense. Another in vitro study showed that TA was able to inhibit the formation of free radicals in mitochondria at key stages of apoptosis [60]. It has also been reported that TA could protect against hydroxyl radical (•OH )-induced oxidative damage in bone marrow-derived mesenchymal stem cells directly through electron transfer, proton-coupled electron transfer, and hydrogen atom transfer, and indirectly through ferrous iron (Fe^2+^) binding [61].

It is worth mentioning that several studies indicated that potent antioxidant approaches, which decrease the CP and other chemotherapy-induced tissue damage, do not interfere with their antitumor activity [62–65]. For instance, one of the clinically approved agents for the prevention of doxorubicin-induced cardiac injury, the iron-chelating agent dexrazoxane, is considered a powerful antioxidant [66]. Hence, it is not likely that the antioxidant role of TA would interfere with chemotherapeutic efficacy of CP. Moreover, TA by itself has also been shown to exhibit a multitude of antitumor properties, including inhibition of the cancer cell proliferation, suppression of stemness by downregulating the protein expression of SOX2 and OCT4, and promotion of the G1 cell cycle arrest and induction of apoptosis, downregulation of the expression of AKT serine/threonine kinase 1 (AKT), among others [67–69]. A previous study showed that TA did not interfere with the cytotoxicity of cisplatin and the combined treatment showed a stronger antiproliferative effect [48]. Furthermore, TA glycoside acted as a potential immunosuppressant agent by exhibiting inhibitory effects against dendritic-cell-mediated immune responses, suggesting that it might be used alone or in combination with other immunosuppressant drugs for the treatment of dendritic-cell-mediated atopic dermatitis [70].

**Figure 8.**
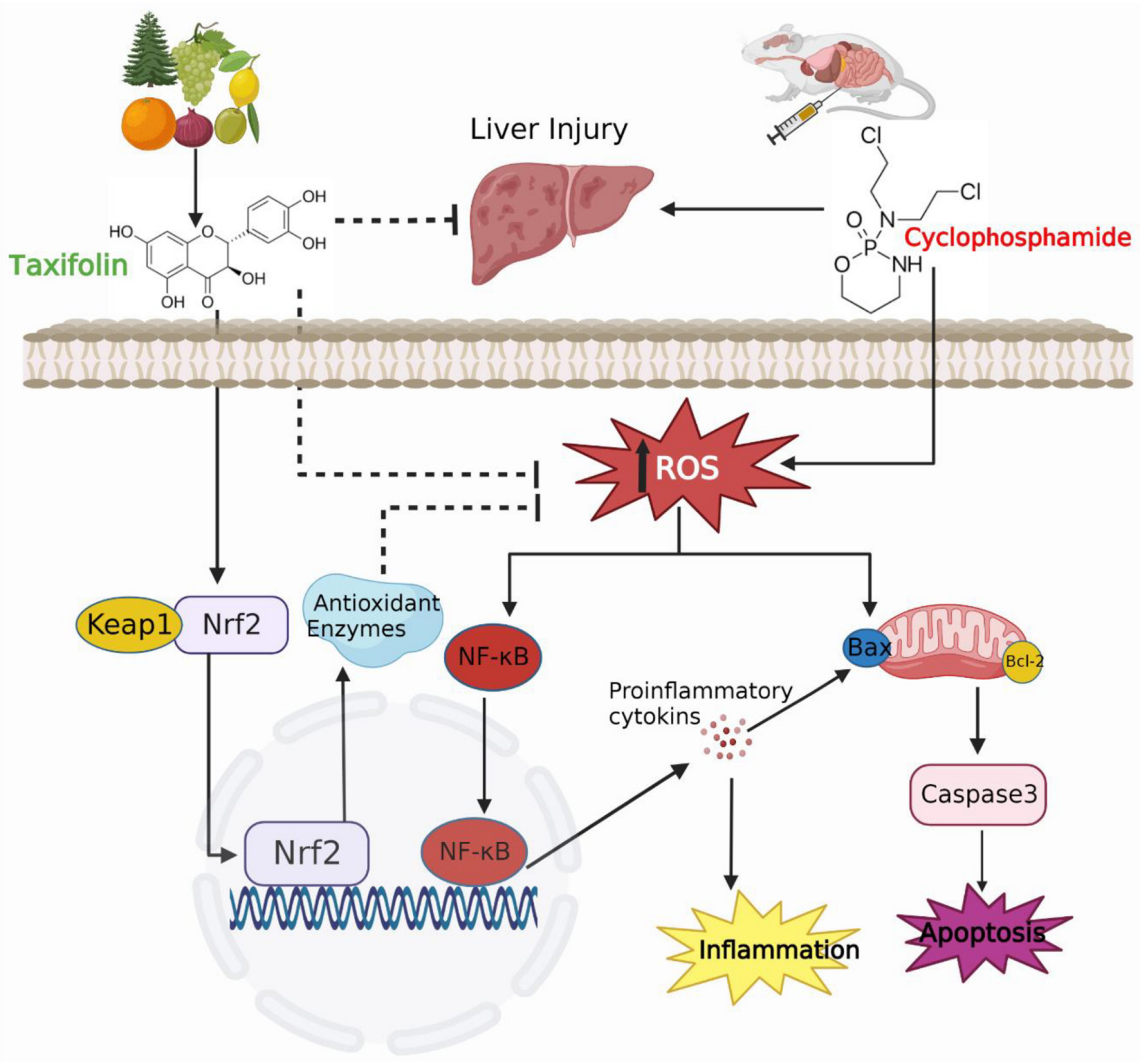
**A schematic summary of CP-induced hepatotoxicity and the hepatoprotective role of TA in mice.** TA was effective in improving cellular redox status, activating Nrf2/HO-1 pathway, mitigating inflammation, and suppressing apoptosis in the liver. CP: Cyclophosphamide; TA: Taxifolin; Nrf2: Nuclear factor erythroid 2-related factor 2; HO-1: Heme oxygenase 1; ROS: Reactive oxygen species; NF-κB: Nuclear transcription factor kappa-B.

It is well-established that increased oxidative stress associated with an impaired antioxidant defense status may activate inflammatory and cell apoptosis signaling pathways, eventually culminating into tissue injury, with subsequent liver dysfunction [5, 6, 9, 34, 71]. Indeed, the CP-induced elevated ROS production may enhance NF-κB activation which in turn induces the production of proinflammatory mediators, leading to a concerted activation of caspase-dependent apoptotic cell death in the liver [5, 9, 22, 72]. This is consistent with our present study demonstrating significantly decreased Bcl-2 expression and activated NF-κB p65, Bax, and caspase-3 expression and increased TNF-α, IL-1β, and IL-6 levels in the liver. In addition to their role in initiating an acute phase of inflammatory response, proinflammatory cytokines promote apoptosis, steatosis, and fibrosis in the liver, aggravating liver dysfunction [73]. Moreover, ROS can mediate apoptosis through mechanisms including the regulation of the expression of various pro-apoptotic proteins, such as Bax and caspases or anti-apoptotic proteins, such as Bcl-2, receptor activation, and mitochondrial dysfunction [74, 75]. Increased hepatic apoptosis is likely to be induced by excessive ROS production that facilitates mitochondrial membrane potential dissipation followed by the release of cytochrome *c* into the cytosol. Cytochrome *c* forms a complex with apoptosis activating factor-1 (Apaf-1) and procaspase-9, called the apoptosome, which in turn results in auto-activation of caspase-9, eventually culminating in cleavage of cellular proteins, DNA fragmentation, and cell demise by apoptosis through activation of executioner caspase-3 [6, 76, 77]. Therefore, suppression of oxidative stress and consequent attenuation of proinflammatory and cell death pathways can be of significant therapeutic benefit.

In the current study, treatment of CP-intoxicated mice with TA suppressed NF-κB p65 expression and reduced TNF-α, IL-1β, and IL-6 levels in the hepatic tissues, indicating additional cytoprotective effects of TA by its anti-inflammatory effects. Likewise, TA ameliorated hepatic expressions of the key apoptotic proteins, including Bax, caspase-3, and Bcl-2, in CP-administrated mice. Consistently, a previous study showed that TA was able to attenuate inflammatory response and apoptotic cell death by suppressing hepatic NF-κB, TNF-α, IL-1β, IL-6, Bax, Bcl-2, and caspase-3 in a mouse model of acute alcohol-induced liver injury [36]. A recent study has also demonstrated that TA mitigated inflammation and apoptosis in CCl_4_-induced hepatic injury in mice by attenuating TNF-α, IL-1β, IL-6, Bax, Bcl-2, and caspase-3 [50]. Besides, it has been demonstrated that TA protected against isoproterenol-induced inflammatory response and apoptosis in the heart by increasing Bcl-2 protein expression and decreasing protein expression levels of NF-κB p65, TNF-α, IL-1β, Bax, and caspase-3 in mouse heart [32]. TA exerted potent suppressive effects on inflammatory and apoptosis signaling pathways in the liver and brain of a rat model of thioacetamide-induced hepatic encephalopathy through downregulation of NF-κB, TNF-α, IL-1β, caspase-3, and Bax expressions and upregulation of IL-10 and Bcl-2 expressions [78]. TA was also shown to prevent iron overload-induced apoptosis by decreasing caspase-3 activity and to improve hepatocellular survival by activation of the prosurvival signaling PI3K/AKT in rats [79]. Taken together, our findings clearly indicate that TA can effectively protect against CP-induced hepatic inflammation and apoptosis, which could possibly be linked to its antioxidant and radicals scavenging activity.

To get a complete picture of the molecular mechanisms underlying the protective effect of TA, we further evaluated the effect of TA treatment on Nrf2/HO-1 signaling pathway since its activation plays a major role in the defense against CP hepatotoxicity [5, 6, 22]. The importance of Nrf2/HO-1 signaling pathway in protection against drug-induced liver injury is supported by a previous published research where Nrf2-deficient mice were highly susceptible to acetaminophen hepatotoxicity and died of liver failure when administered with acetaminophen doses that were tolerated by wild-type mice [80]. Indeed, Nrf2 is an important transcription factor that controls the basal and stress-inducible activation of a number of antioxidant and detoxification genes [81, 82]. Moreover, Nrf2 suppresses the NF-κB-mediated inflammatory response through mitigating oxidative stress-mediated NF-κB activation and preventing the IκB-α proteasomal degradation and consequently inhibiting nuclear translocation of NF-κB [83, 84]. Based on that, Nrf2/HO-1 activation is considered as a potential therapeutic target for the treatment of drug-induced oxidative damage and organ failure. In agreement with previous studies [5, 6], the findings of this study demonstrated that CP injection resulted in Nrf2 and HO-1 downregulation in the liver. On the other hand, the current study found that treatment of CP-injected mice with TA (25 mg/kg) effectively decreased oxidative damage and restored liver Nrf-2/HO-1 to normal levels, whereas treatment with a higher dose of TA (50 mg/kg) caused significant activation and upregulation of Nrf-2/HO-1, indicating that TA may play a role in activation and upregulation of Nrf-2/HO in a dose-dependent manner. These findings are in agreement with previous reports where Nrf2/HO-1 activation mediated the protective effect of TA against isoproterenol-induced cardiac oxidative stress and inflammation [32] and bacterial endotoxin lipopolysaccharide-induced increased inflammation and mortality [49] in mice. Hence, it can be speculated that Nrf2/HO-1 activation may contribute to the antioxidant and anti-inflammatory properties of TA in CP-injected mice.

## Conclusion

The findings of this study showed that treatment with the natural product TA can attenuate CP-induced liver injury by decreasing the extent of oxidative tissue injury, inflammatory response, and hepatocyte apoptosis. These data also indicated that the beneficial effects of TA were associated with the activation of Nrf2/HO-1 and restoration of cellular redox status in the liver (Figure 8). Thus, TA may have tremendous therapeutic potential in the prevention/treatment of CP hepatotoxicity and perhaps a multitude of diseases associated with oxidative stress and inflammation, which warrants future clinical studies.
